# Association of Insulin-like Growth Factor 1 Concentrations with Risk for and Prognosis of Amyotrophic Lateral Sclerosis – Results from the ALS Registry Swabia

**DOI:** 10.1038/s41598-020-57744-x

**Published:** 2020-01-20

**Authors:** Gabriele Nagel, Raphael S. Peter, Angela Rosenbohm, Wolfgang Koenig, Luc Dupuis, Dietrich Rothenbacher, Albert C. Ludolph

**Affiliations:** 1 Institute of Epidemiology and Medical Biometry, Ulm University, Ulm, Germany; 2 Department of Neurology, Ulm University, Ulm, Germany; 3 Deutsches Herzzentrum München, Technische Universität München, Munich, Germany; 4 DZHK (German Centre for Cardiovascular Research), partner site Munich Heart Alliance, Munich, Germany; 5 INSERM U1118, Université de Strasbourg, Strasbourg, France

**Keywords:** Prognostic markers, Risk factors

## Abstract

We investigated the associations of serum concentration of insulin-like growth factor 1 (IGF1) with risk and prognosis of ALS in the ALS registry (October 2010–June 2014, median follow-up 67.6 months) in a case-control and cohort study, respectively. Serum samples were measured for IGF-1. Information on covariates was collected by standardized questionnaire. We applied conditional logistic regression to appraise the risk and Cox proportional hazards models to appraise the prognostic value of IGF-1. Data of 294 ALS patients (mean age 65.4 (SD 11.0) years, 60.2% men) and 504 controls were included in the case-control study. Median serum IGF-1 concentrations were slightly higher in ALS cases than in controls (101 vs. 99.5 ng/ml). IGF-1 concentrations were not associated with ALS risk in the fully adjusted model (top vs. bottom quartile: OR 1.16; 95%-CI 0.73–1.84, p for trend = 0.44). Among 293 ALS cases (mean age 65.5 (SD 10.5) years, 56.8% men) 243 died during follow-up. We found a statistically significant inverse association between continuous IGF-1 concentrations and survival (p = 0.01). Very high values IGF-1 were associated with a better prognosis of ALS suggesting that functions related to IGF-1 could be involved in survival.

## Introduction

Amyotrophic lateral sclerosis (ALS) is a neurodegenerative disease of largely unknown etiology leading to death within 3–4 years after diagnosis^1^. There is growing evidence that a disturbed energy metabolism in ALS could play a pathogenic role^2^. Data from the ALS registry Swabia showed a possible positive association of body mass index (BMI) with ALS decades before the clinical manifestation of ALS^3^. In the ALS cases, there was a sharp kink in BMI trajectories shortly before onset of ALS, and greater weight loss was associated with a worse prognosis^3^. A further investigation has shown that serum concentrations of leptin and adiponectin, two key hormones of the energy metabolism, are strongly and independently of the BMI associated with the ALS risk. Serum leptin levels were also negatively associated with overall survival of ALS patients^4^.

Insulin-like growth factor 1 (IGF-1) is a pluripotent growth factor with multiple functions in the peripheral and central nervous system. It supports neuronal survival and axon growth^5,6^. IGF-1 was found to protect the motoneurons^7^. In ALS patients plasma IGF-1 levels were reduced, suggesting that the Growth hormone (GH)/IGF-1 axis could be a serological marker of some specific neuronal degeneration^8^. The results of experimental studies indicate that low IGF-1 concentrations could play a role in the course of ALS disease by accelerating neurodegenerative changes and worsening clinical symptoms^9^. Research in mice suggested that IGF1-related mitochondrial protection might also play a role^10^. Increasing muscle derived IGF-1 was found protective in mouse models of ALS^11^, and delivering IGF-1 in the CNS also led to increased survival^12,13^. Understanding the IGF-1 system in neurons, including the regulation of IGF-1 availability and signaling mechanisms, could be an important aspect for development of ALS therapeutic approaches^14^.

The objective of this study was to analyze the associations of serum IGF-1 concentrations with the risk of ALS in a population-based case-control study. Furthermore, we investigated the association of IGF-1 serum concentration with prognosis of ALS in a cohort-design in ALS-cases only.

## Material and Methods

### Study design and study population

The ALS registry Swabia has been described previously in detail^15–17^. In brief, it is a population-based clinical-epidemiological registry with the aim to collect data on all newly diagnosed ALS cases in Swabia, a defined geographic region with approximately 8.4 million inhabitants in the South-West of Germany.

All reported ALS cases were defined by the diagnosis of possible, probable or definite ALS according to the revised El Escorial criteria by an experienced neurologist. Notifications of patients with suspected ALS were tracked and evaluated during the clinical course by the registry.

Patients prospectively registered between October 01, 2010 and June 30, 2014 were asked to provide informed consent to participate in a population-based case-control study. For each case (N = 289), two sex and age frequency-matched control subjects (N = 504) were randomly selected from the general population as registered in the regional registry office (“Einwohnermeldeamt”). The thus identified subjects were contacted by postal mail and invited to participate in the study. After written informed consent was obtained, study nurses visited the participants for a standardized interview and convenience blood sampling. Median time since the last meal was 3.2 hours in ALS cases and 3.2 hours in controls. The standardized instruments and tests were performed identically in ALS-cases and controls. On population-level in the target population in Swabia, response in cases was 65% (20% refused and 15% could not been contacted) and in the population-based controls 19% (39% refused and 42% did not respond after several attempts to get in contact per mail and telephone).

In addition, ALS cases were actively followed-up and interviewed on a yearly basis. To update vital status record linkage with the central registration office in Baden-Württemberg and the local registration offices in Bavaria were performed (last update October 16^th^, 2018). In case of death, the date of death was obtained from the local registration office.

### Ethics statement

International, national and state rules were followed implementing the ALS registry Swabia. We obtained full ethical approval of the ethical committees of the Ulm University and the regional physician chambers (“Landesaerztekammer Baden-Wuerttemberg” and “Landesaerztekammer Bayern”).

### Laboratory measurements

According to a common standard protocol for cases and controls, blood samples were transported in cooled containers to the study center. Serum was obtained by centrifugation for 10 min at 2000 RPM × g and 4 °C (Heraeus Multifuge 3 S-R, Fa. Thermofischer). Blood specimens were transferred into 0.5–1.0 ml aliquots with screw tops on the same day and stored at −80° Celsius until further analysis. IGF-1 (ng/mL) was measured in serum (human IGF-1 Quantikine, R&D Systems). The lower detection limit of IGF-1 in this assay was approximately 0.026 ng/mL. The inter-assay coefficient of variation (CV) was 12.5%. Further details can be found in the product data sheet of the manufacturer. All laboratory analyses were performed in blinded fashion at the Biomarker Laboratory of the Department of Internal Medicine II-Cardiology, Ulm University Medical Center.

### Statistical methods

Conditional logistic regression was used to calculate multivariable odds ratios (OR) and 95% confidence intervals (CI) for the association of ALS with quartiles of serum IGF-1 concentrations. Quartile cut-points were calculated based on the distribution in controls. Models were stratified for age groups and adjusted for duration of school education, occupational work intensity, smoking (ever, never), family history of ALS, BMI, and diabetes mellitus. The models are based on data with full set of covariates. Beside quartiles we also split the top quartile into two categories (75– < 90^th^ and ≥90^th^ percentile) to further investigate associations with more extreme values.

In the cohort part Cox proportional hazards models adjusted for age, diagnostic delay, site of onset and ALS-functional rating scale-revised (FRS-R) were applied to calculate hazard ratios (HRs) for overall survival. Survival times were censored at the date of the last systematic mortality update (October 16^th^, 2018). In addition, we used cubic restricted splines (with knots at 10, 50, and 90% percentiles) on IGF-1 as continuous variable to model a possible nonlinear association with mortality. Sensitivity analyses excluding the El Escorial categories “clinically suspected” and “clinically possible” were performed in the adjusted model.

We calculated ALS-FRS-R decline, as (48 - ALS-FRS-R at interview) divided by (time from disease onset to interview in month), as marker of disease progression. The association of IGF-1 and ALS-FRS-R decline was modeled using cubic restricted splines (with knots at 10, 50, and 90% percentiles) adjusted for age, sex and time since last meal.

All provided p-values are two-sided. The statistical software package SAS release 9.4 (SAS Institute, Cary, NC, USA) was used.

## Results

The case-control study comprised 294 ALS cases (60.2% men) with a mean age of 65.4 (SD 11.0) years and 504 controls (59.3% men) with a mean age of 66.3 (SD 10.3) years **(**Table 1**)**. Compared to controls, ALS patients were characterized by lower school education, BMI, occupational work intensity, respectively and median concentration of serum IGF-1 were slightly higher in ALS cases than in controls (101.0 vs. 99.7 ng/ml). Most ALS cases showed lumbar (34.0%), bulbar (31.0%) or cervical (25.5%) onset. According to the revised El Escorial criteria, more than 70% of the ALS cases had a probable or definite diagnosis.

**Table 1:** Main characteristics of ALS patients and control subjects.

	N_Cases_	ALS-cases	N_Controls_	Control subjects
Age (years), mean (SD)	294	65.4 (11.0)	504	66.3 (9.8)
Sex	294		504	
Male, N (%)		177 (60.2)		299 (59.3)
School education, N (%)	294		501	
<10^th^ grade		163 (55.4)		226 (45.1)
≥10^th^ grade		131 (44.7)		275 (54.9)
Smoking	291		503	
Ever, N (%)		134 (46.1)		242 (48.1)
BMI (kg/m^2^), mean (SD)	294	24.6 (4.1)	502	26.5 (4.0)
<23, N (%)		110 (37.4)		86 (17.1)
23 – <25, N (%)		66 (22.5)		113 (22.5)
25 – <28, N (%)		63 (21.4)		151 (30.1)
≥28, N (%)		55 (18.7)		152 (30.3)
Family history of ALS, N (%)	290		504	
Positive		12 (4.1)		2 (0.4)
Occupational work intensity, N (%)	283		498	
Light (mainly sitting)		102 (36.0)		231 (46.4)
Moderate (standing and walking)		116 (41.0)		203 (40.8)
Heavy (physically demanding)		65 (23.0)		64 (12.9)
Diabetes mellitus prevalence, N (%)	285	26 (9.1)	492	54 (11.0)
IGF-1 (ng/ml), median (Q1,Q3)	294	101.0 (81.2, 123.0)	504	99.5 (80.7, 120.0)
Time since last meal before blood sampling (h), median (Q1,Q3)	284	3.2 (0.5, 18.5)	487	3.2 (0.25, 17.5)
Time of blood sampling (hh:mm), median (Q1, Q3)	288	11:00 (10:00, 12:30)	496	12:30 (11:30, 13:45)
*Clinical characteristics of ALS-cases*				
Site of onset, N (%)	294			
Bulbar		91 (31.0)		
Cervical		75 (25.5)		
Thoracic		13 (4.4)		
Lumbar		100 (34.0)		
Uncertain		15 (5.1)		
Revised El Escorial criteria, N (%)	289			
Clinically suspected		47 (16.3)		
Clinically possible		36 (12.5)		
Clinically probable		95 (32.9)		
Clinically probable – lab. supported		81 (28.0)		
Clinically definite		30 (10.4)		
ALS-FRS, median (Q1, Q3)	293	39.0 (34.0, 43.0)		
Diagnostic delay (month), median (Q1, Q3)	294	5.0 (2.7, 9.0)		
Diagnosis to baseline visit (month), median (Q1, Q3)	294	3.6 (2.1, 6.0)		

The comparison between ALS cases and controls revealed differences in school education for the category 10 years or more (44.7% vs. 54.9%) and heavy occupational work intensity (23.0% vs. 12.9%), and prevalence of diabetes mellitus which was reported by 9.1% of ALS cases and 11.0% of controls. Compared to participants until the age of 65 years, older age was associated with lower IGF-1 concentrations (p-value < 0.001, Supplemental Table 1).

In the fully adjusted model **(**Table 2**)**, IGF-1 serum concentrations were not associated with the risk of ALS neither in crude nor in fully adjusted models (top vs. bottom quartile: OR 1.12; 95% CI 0.71,1.77, p for trend 0.53). However, when the upper quartile was further divided, increased ALS risk for increased IGF-1 concentrations become somewhat more prominent (90^th^ percentile vs. bottom quartile: OR 1.38; 95% CI 0.79–2.43, p for trend 0.37), however, the 95% CI of the point estimate included the null-effect value. By using restricted cubic splines and IGF-1 serum concentrations as a continuous measure we did not find evidence for an association with ALS risk (p-value 0.21, Supplemental Fig. 1). Sensitivity analyses with “clinically suspected” and “clinically possible” cases only suggested an increased point estimate for top vs. bottom quartile with an OR of 1.24 (p for trend 0.33), but the 95% CI from 0.73 to 2.09 was wide and also included the null-effect values (Supplemental Table 2).

**Table 2:** Odds ratios for ALS by quartiles of serum IGF-1 concentrations.

	Odds ratio (95%-CI)
Crude (N_Cases_ = 294, N_Controls_ = 504)^a^	
Bottom quartile (<80.8 ng/ml)	(ref.) 1.00
2^nd^ quartile (80.8 –<99.9 ng/ml)	0.91 (0.60, 1.38)
3^rd^ quartile (99.9 –<121 ng/ml)	1.06 (0.70, 1.60)
Top quartile (≥121 ng/ml)	0.96 (0.63, 1.45)
p-value for trend	0.97
Adjusted (N_Cases_ = 277, N_Controls_ = 494)^b^	
Bottom quartile (<80.8 ng/ml)	(ref.) 1.00
2^nd^ quartile (80.8 –<99.9 ng/ml)	0.93 (0.60, 1.42)
3^rd^ quartile (99.9 –<121 ng/ml)	1.09 (0.71, 1.69)
Top quartile (≥121 ng/ml)	1.09 (0.71, 1.67)
p-value for trend	0.56
Add. adj. for diabetes and BMI (N_Cases_ = 260, N_Controls_ = 464)^c^	
Bottom quartile (<80.8 ng/ml)	(ref.) 1.00
2^nd^ quartile (80.8 –<99.9 ng/ml)	0.91 (0.57, 1.45)
3^rd^ quartile (99.9 –<121 ng/ml)	1.02 (0.64, 1.62)
Top quartile (≥121 mg/l)	1.12 (0.71, 1.77)
p-value for trend	0.53
*75-* < 90^*th*^ *percentile (121-* > *140* ng/ml)	*0.94 (0.54–1.62)*
90^*th*^ *(percentile* ≥*140* ng/ml)	*1.38 (0.79, 2.43)*

^a^Controlled for age and sex. ^b^Additionally adjusted for school education, occupational work intensity, smoking (ever) and family history of ALS. ^c^As ^b^but additionally adjusted for diabetes, body mass index (BMI), and time since last meal.

In the cohort part, which included the ALS-cases only, 243 deaths in 293 cases were identified during a median follow-up of 67.6 months. Table 3 shows the characteristics of these patients. Median IGF-1 concentration was 52.0 ng/ml in deceased compared to 55.0 ng/ml in ALS patients who survived.

**Table 3:** Characteristics of ALS patients (N = 293) with mortality follow-up by survival status.

	N_Deceased_	Deceased	N_Survived_	Survived
Age (years), mean (SD)	243	66.5 (10.5)	50	60.5 (11.7)
Sex	243		50	
Male, N (%)		138 (56.8)		38 (76.0)
BMI (kg/m^b^), mean (SD)	243	24.5 (4.0)	50	24.8 (4.5)
Diagnostic delay (month), median (Q1, Q3)	243	5.0 (2.7, 8.5)	50	6.0 (2.5, 11.1)
ALS-FRS, median (Q1, Q3)	242	38.5 (34, 42)	50	41 (38, 45)
IGF-1 (ng/ml), median (Q1, Q3)	243	101.0 (82.2,118.0)	50	105.5 (74.6, 137.0)

**Figure 1:**
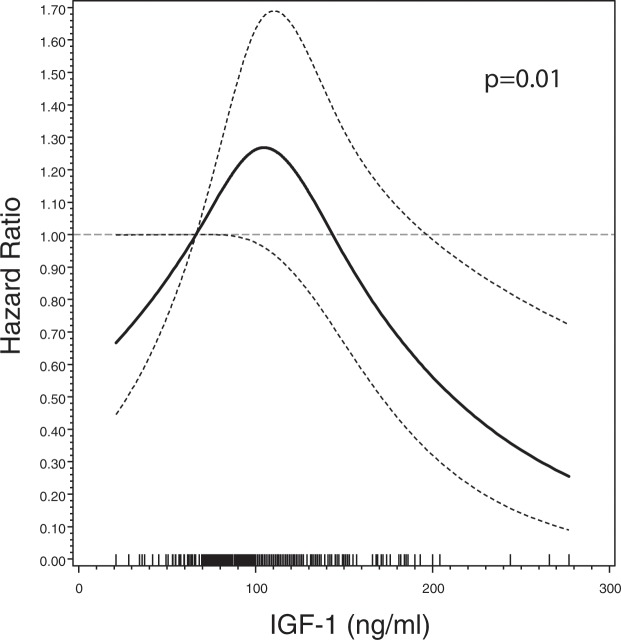
Serum IGF-1 concentration and HR (95% confidence band) of ALS mortality among 293 ALS patients by cubic splines, adjusted for age, sex, diagnostic delay, site of onset, ALS-FRS, and body mass index. The dashed grey line represents the reference value (HR = 1 at 66.3 ng/ml). Bottom rugs represent measured IGF-1 values.

The evaluation of IGF-1 in quartiles revealed no clear pattern of the associated HR, i.e. the HR corresponding to the top vs. bottom quartile was 0.90 (95% CI; 0.61–1.31, Table 4). The top quartile has been divided in the 75– < 90^th^ and ≥90^th^ percentiles in order to analyze more refined association in the top quartile. The splitting revealed an HR of 0.87 (95%CI 0.54–1.40) and HR of 0.93 (95% CI 0.58–1.48), respectively. However, the evaluation of the prognostic value of IGF-1 serum concentrations as a continuous measure by using restricted cubic splines revealed a nonlinear association with reduced mortality for high IGF-1 levels (p-value 0.01, Fig. 1**)**. In addition, IGF-1 serum concentrations were in a similar nonlinear manner associated with ALS-FRS-R decline assessed at baseline (Supplemental Fig. 2, p = 0.02).

**Table 4:** Hazard ratio by quartiles of serum IGF-1 concentrations among ALS patients.

	Hazard ratio (95%-CI)
Crude (N_Deceased_ = 243, N_Survived_ = 50)^a^	
Bottom quartile (<80.8 ng/ml)	(ref.) 1.00
2^nd^ quartile (80.8 -<99.9 ng/ml l)	1.29 (0.90, 1.85)
3^rd^ quartile (99.9 -<121 ng/ml)	1.17 (0.82, 1.66)
Top quartile (≥121 ng/ml)	0.97 (0.67, 1.40)
p-value for trend	0.78
Adjusted (N_Deceased_ = 242, N_Survived_ = 50)^b^	
Bottom quartile (<80.8 ng/ml)	(ref.) 1.00
2^nd^ quartile (80.8 –<99.9 ng/ml)	1.13 (0.78, 1.64)
3^rd^ quartile (99.9 –<121 ng/ml)	1.22 (0.84, 1.76)
Top quartile (≥121 ng/ml)	0.90 (0.61, 1.31)
p-value for trend	0.66
75– < 90^*th*^ *percentile (121-*>*140* ng/ml)	0.87 (0.54, 1.40)
90^*th*^ *percentile* (*≥140* ng/ml)	0.93 (0.58, 1.48)

^a^Controlled for age and sex. ^b^Additionally adjusted for diagnostic delay, site of onset, ALS-FRS and body mass index.

## Discussion

In this population-based case-control study in Southern Germany, serum IGF-1 concentrations were not associated with risk of ALS. In the cohort of ALS patients, however, we found evidence for an inverse association between high serum IGF-1 concentrations and overall survival.

Our results in the ALS cohort concerning prognosis are in line with observations of others. In ALS patient with longer survival, peripheral IGF-1 concentrations were increased by 58% compared to controls^18^, suggesting that higher IGF-1 concentrations may be associated with favorable survival. Beauverd *et al*. (2012) performed a meta-analysis including 779 patients from three studies, which, however, the authors saw as seriously compromised by details of trial designs^19^. The pooled results, however, of two trials suggested that treatment with recombinant human IGF-1 might be beneficial, but in one study no benefit in survival was found^19^. In addition, our observation that higher IGF-1 concentration is associated with longer survival is consistent with experimental research, showing that IGF-1 acts as a mitochondrial protector in the ALS cell and mouse model^10^. A results of a recent trial on IGF-1 in the treatment of spinal and bulbar muscular atrophy (SBMA), a rare motoneuron disease of the peripheral muscle with slow progression, did not improve muscle strength or function^20^.

Indeed, higher circulating concentrations of IGF-1 were related to increased survival in ALS mouse model^21^. IGF-1 and its binding proteins were also affected in muscles of ALS patients^22^. Importantly, IGF-1 led to increased survival of ALS patients in most^11–13,23^ but not all^24^ studies.

Multiple mechanisms elicited by IGF-1 might account for the observed increased survival in patients with higher circulating IGF-1. IGF-1 displays high neurotrophic properties, which could protect motor neurons and increase survival in ALS^25^. IGF-1 has also anabolic actions on skeletal muscle, especially upon denervation^26^, which might be beneficial in ALS. Indirectly, increased circulating IGF-1 could be a fingerprint of higher GH-IGF1 axis activity, and GH is able to modulate oligodendroglial cell survival and myelination in the CNS^27^. Interestingly growth hormone secretion was dysregulated in both patients and mouse models of ALS^28^. Results from experimental studies indicate a circadian control of IGF-I production^29,30^. In our study the time of blood sampling was in median about 1.5 hours earlier in ALS cases than in control subjects, with large overlap in the distribution. Furthermore, it can be speculated whether atrophy of the hypothalamus in ALS patients is related to the different IFG-I levels^31^.

Since IGF-1 is also related to energy metabolism and body weight^32^, our current findings are consistent with former observations concerning BMI and adipokines as well as the findings on retinol binding protein (RBP)4 and the prognosis of ALS^3,19,33^. Importantly, however, the observed protective effect of higher IGF-1 was observed upon adjustment of BMI, suggesting that it is not fully mediated by BMI as marker for fat mass. Thus, the longer survival of patients with higher IGF-1 levels could be related to direct biological actions of IGF-1, to an overactivation of a protective GH-IGF-1 pathway or to altered IGF-1 levels in patients with a favorable metabolic status.

Strengths of our study are its relatively large sample size and the virtually complete follow-up of the ALS patient cohort^17^. In addition, in the case-control part we carefully matched for sex, age as well as geographic region and used multivariable analysis to further adjust for potential confounders such as physical activity, levels of education and smoking status. Yet, adjustment for diabetes and BMI did not substantially change the risk estimates. In addition, IGF-1 was measured according to a standard protocol under blinded conditions.

However, there are also limitations which need to be considered. Residual confounding cannot be ruled out. In addition, in order to account for the blood sampling at convenience we adjusted for the time since last meal in the analyses. When generalizing the results of the case-control study the low participation rate among the controls should be considered. However, IGF-1 as prognostic factor was analyzed in a cohort of ALS patients with median follow-up of 67.6 months which is representative for the entire ALS registry Swabia^17^. Furthermore, when looking on the results we have to keep the limited sample size in both study parts in mind. The power of the case-control study was insufficient to detect a weak association between IGF-1-serum concentrations and risk of ALS. It was about 80% to detect an OR of 1.8 (i.e. a minimum risk increase of 80%) at α = 0.05 associated with the upper quartile compared to the bottom one. In the cohort part, the power to detect was HR 0.6 (i.e. a risk reduction of 40%) under the same conditions.

In summary, our study showed no clear association of serum IGF-1 concentration with ALS risk but with the prognosis of ALS, suggesting that higher IGF-1 concentration could increase survival. Further research including a prospective design and other biological markers is necessary to clarify the role of IGF-1 in disease progression of ALS.

## Supplementary information

Supplemental material.

## Data Availability

Due to ethical restrictions regarding data protection issues and the study specific consent text and procedure, the data cannot be made publicly available, but data are available to all interested researchers upon request.
