# Supplementary material for: Association of Insulin-like Growth Factor 1 Concentrations with Risk for and Prognosis of Amyotrophic Lateral Sclerosis – Results from the ALS Registry Swabia
Source: Sci Rep. 2020 Jan 20;10:736. doi: 10.1038/s41598-020-57744-x (PMC6971302; doi:10.1038/s41598-020-57744-x)

**Supplement** **to**

**Association of Insulin-like Growth Factor 1 Concentrations with Risk for and Prognosis of Amyotrophic Lateral Sclerosis – Results from the ALS Registry Swabia**

Gabriele Nagel^1^, Raphael S Peter^1^, Angela Rosenbohm^2^, Wolfgang Koenig ^3,4^, Luc Dupuis^5^, Dietrich Rothenbacher^1^, Albert C Ludolph^2^

**Supplemental Table 1.** Association of selected sociodemographic and clinical variables with Insulin Growth Factor (IGF-1)

|  |  |  |  | **Insulin Growth Factor (ng/ml)** | |
| --- | --- | --- | --- | --- | --- |
|  |  | **N** |  | **geometric mean** | **p-value** |
| Age (years) | |  |  |  |  |
|  | < 65 | 340 |  | 106.2 |  |
|  | ≥ 65 | 458 |  | 92.2 | ^a^<.0001 |
| Sex | |  |  |  |  |
|  | Female | 322 |  | 96.0 |  |
|  | Male | 476 |  | 99.2 | ^b^0.24 |
| BMI (kg/m^2^) | |  |  |  |  |
|  | <23 | 196 |  | 96.1 |  |
|  | 23 - <25 | 179 |  | 101.6 |  |
|  | 25 - <28 | 214 |  | 99.0 |  |
|  | ≥28 | 207 |  | 95.5 | ^c^0.18 |
| School education | |  |  |  |  |
|  | < 10^th^ grade | 389 |  | 95.4 |  |
|  | ≥ 10^th^ grade | 406 |  | 100.3 | ^c^0.18 |
| Smoking | |  |  |  |  |
|  | Never | 418 |  | 99.5 |  |
|  | Ever | 376 |  | 97.1 | ^c^0.21 |
| Occupational work intensity | |  |  |  |  |
|  | Light | 333 |  | 99.1 |  |
|  | Moderate to heavy | 448 |  | 97.4 | ^c^0.40 |
|  |  |  |  |  |  |

^a^ adjusted for case-control status, and sex
^b^ adjusted for case-control status, and age
^c^ adjusted for case-control status, sex and age

**Supplemental Figure 1.** Serum IGF-1 concentration and OR (95% confidence band) of ALS risk among 260 ALS cases and 464 controls by cubic splines, adjusted for age, sex, school education, occupational work intensity, smoking (ever), family history of ALS, diabetes, body mass index (BMI), and time since last meal. The reference value of OR = 1 is at 66.3 ng/ml. Bottom rugs represent measured IGF-1 values.

**
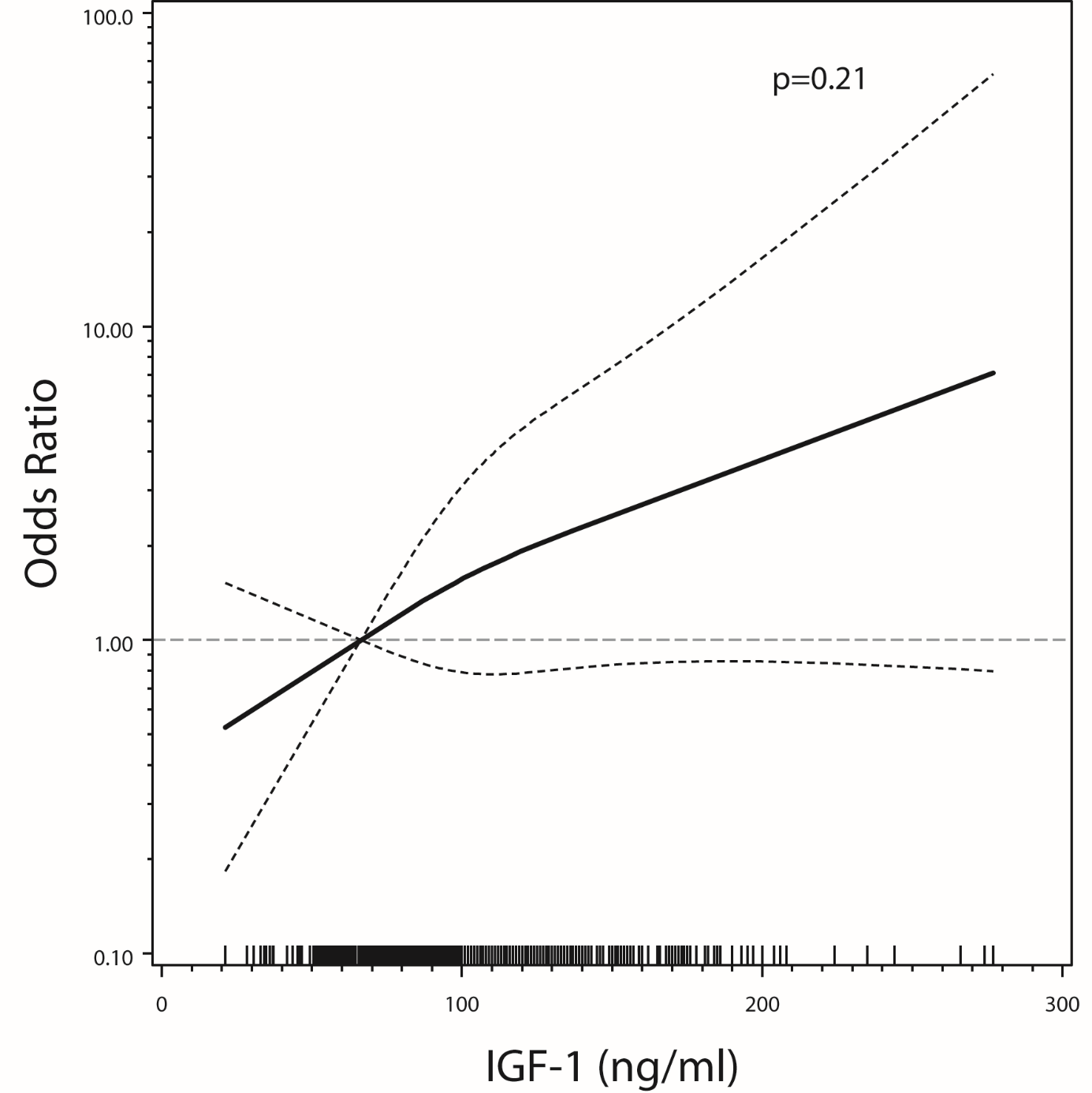
**

**Supplemental Table 2**. Odds ratios for ALS by quartiles of serum IGF-1 concentrations Sensitivity analysis for "clinically suspected" and "clinically possible" cases

|  | | **Hazard ratio (95%-CI)** |
| --- | --- | --- |
| Adjusted ^a^ (N_Cases_=180, N_Controls_=464) | |  |
|  | Bottom quartile (<80.8 ng/ml) | (ref.) 1.00 |
|  | 2^nd^ quartile (80.8 - <99.9 ng/ml) | 0.93 (0.54, 1.60) |
|  | 3^rd^ quartile (99.9 - <121 ng/ml) | 1.12 (0.66, 1.90) |
|  | Top quartile (≥121 ng/ml) | 1.24 (0.73, 2.09) |
| p-value for trend | | 0.33 |

^a^ controlled for age and sex and adjusted for school education, occupational work intensity, smoking (ever) and family history of ALS diabetes and body mass index (BMI), time since last meal

**Supplemental Figure 2.** ALS-FRS-R decline per month (95% confidence band) in 260 ALS cases by cubic splines of IGF-1 concentration, adjusted for age, sex, and time since last meal. Bottom rugs represent measured IGF-1 values.


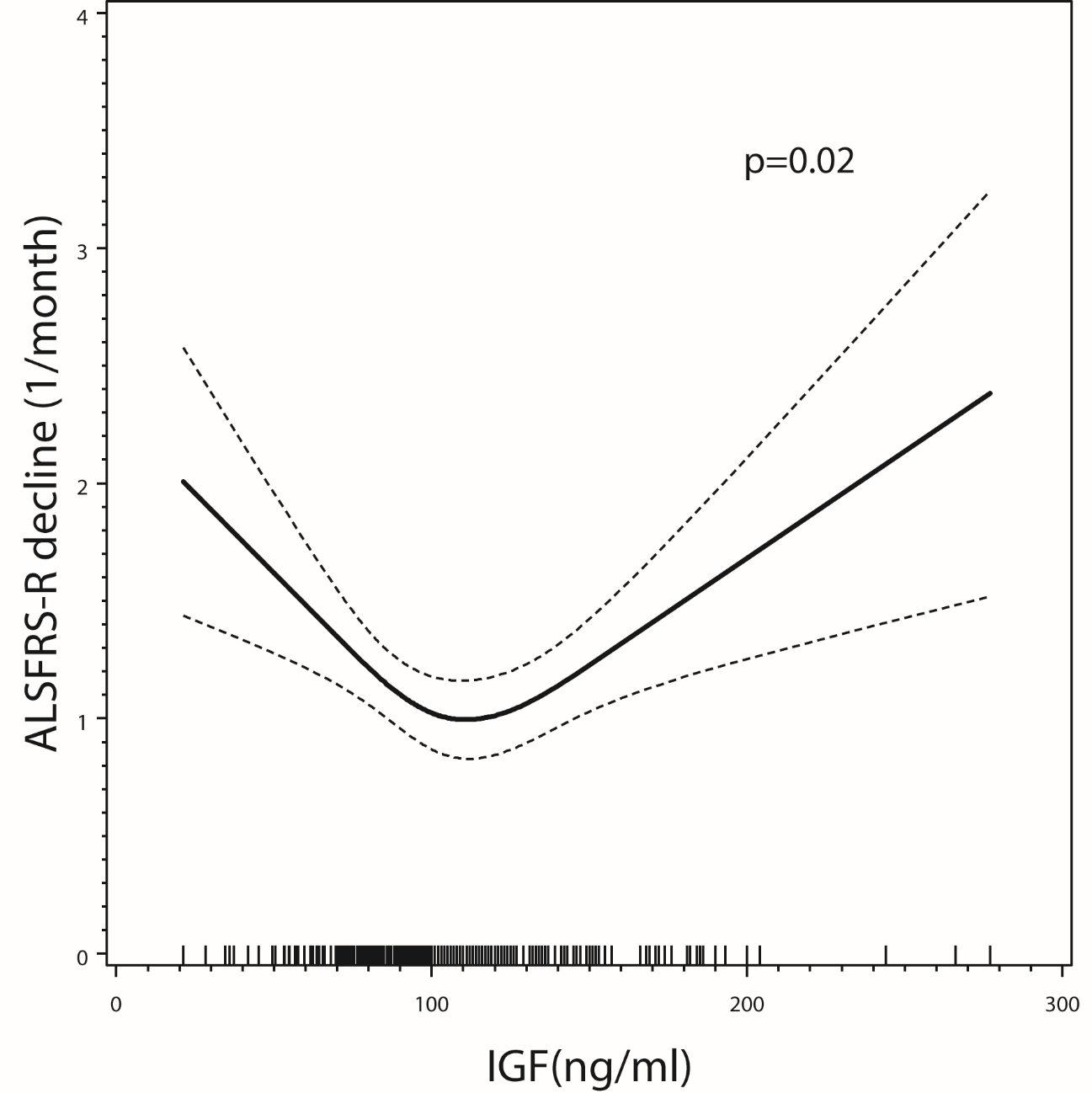

Supplement: Supplementary file 1 — Supplemental material. [file 41598_2020_57744_MOESM1_ESM.docx]
